# One-Time Use of Galcanezumab or Fremanezumab for Migraine Prevention

**DOI:** 10.7759/cureus.34180

**Published:** 2023-01-25

**Authors:** Masahito Katsuki, Kenta Kashiwagi, Shin Kawamura, Senju Tachikawa, Akihito Koh

**Affiliations:** 1 Department of Neurosurgery, Itoigawa General Hospital, Itoigawa, JPN; 2 Department of Neurology, Itoigawa General Hospital, Itoigawa, JPN

**Keywords:** one-time use, headache, migraine, calcitonin gene-related peptide-related monoclonal antibodies, adherance

## Abstract

Background

There are no reports on the effectiveness of one-time use of the calcitonin gene-related peptide-related monoclonal antibodies (CGRP-mABs) evaluated at one and three months for migraine prevention. Here, we present the real-world data of one-time administration of CGRP-mABs, galcanezumab and fremanezumab, for migraine prevention.

Methodology

We retrospectively investigated eight migraine patients treated with one-time administration of galcanezumab 240 mg or fremanezumab 225 mg. Monthly headache days (MHD), monthly acute medication intake days (AMD), and Headache Impact Test-6 (HIT-6) scores before, one, and three months after one-time CGRP-mABs administration were evaluated.

Results

A total of five women and three men were included (median age = 46.5 years, range = 19-63 years). Overall, six were episodic migraine, and two were chronic migraine. Five patients received one-time administration of fremanezumab and three received galcanezumab. In total, six (75.0%) patients experienced therapeutic effectiveness one month after the one-time administration. Five of the six maintained the therapeutic effect until three months, but one had aggravation. As a result, six (75.0%) patients reached or maintained therapeutic conditions three months after the one-time administration of CGRP-mABs without side effects. All patients continued previously used oral prophylaxis during the observational period. Significant reductions in MHD, AMD, and HIT-6 scores were observed three months after the initial administration (p = 0.008, p = 0.005, and p < 0.001, respectively).

Conclusions

Six of the eight patients experienced or maintained therapeutic effectiveness at three months despite the one-time administration of CGRP-mABs. Our results suggest that one-time use of CGRP-mABs may be a new treatment option in combination with oral prophylaxis.

## Introduction

Migraine is a widespread public health problem [1]. In Japan, migraine prevalence is 0.9-8.4% [2-6], and 29.8-74.2% of patients with migraine headaches report that it significantly impairs their daily activity [1]. Galcanezumab and fremanezumab, calcitonin gene-related peptide-related monoclonal antibodies (CGRP-mABs), are used for migraine prevention. In the randomized placebo-controlled trials for galcanezumab [7], EVOLVE-1 (ClinicalTrials.gov identifier: #NCT02614183: Evaluation of Galcanezumab in the Prevention of Episodic Migraine 1) study, EVOLVE-2 (#NCT02614196) study, and REGAIN (#NCT02614261: Evaluation of Galcanezumab in the Prevention of Chronic Migraine) study, the efficacy of galcanezumab was evaluated at one and three months with the continuation of monthly administration throughout the term. In the randomized placebo-controlled trials for fremanezumab [8], the HALO EM study (#NCT02629861: Efficacy and Safety of 2 Dose Regimens of Fremanezumab Versus Placebo for the Preventive Treatment of Episodic Migraine), HALO CM study (#NCT02621931: Comparing Efficacy and Safety of 2 Dose Regimens of Subcutaneous Administration of Fremanezumab Versus Placebo for the Preventive Treatment of Chronic Migraine), and FOCUS (#NCT03308968: An Efficacy and Safety Study of Fremanezumab in Adults With Migraine) study, the efficacy of fremanezumab was evaluated at the 12 weeks. However, there are no reports on the effectiveness of one-time use of the two drugs evaluated at one and three months. Here, we present the real-world data of eight migraine patients treated with one-time administration of galcanezumab 240 mg or fremanezumab 225 mg, as an early clinical experience. Our hypothesis is that one-time use of CGRP-mABs may have a therapeutic effect after three months, for which we retrospectively evaluated such patients. This article was previously posted to the Researchgate preprint server in December 2022.

## Materials and methods

Study population

Inclusion criteria included (1) patients aged 18 to 65 years; (2) patients diagnosed with migraine; (3) patients who had had at least one type of prophylactic medication before CGRP-mABs use, but it did not have sufficient efficacy or adverse effects; (4) patients who suffered headaches at least 90 days before the CGRP-mABs treatment and had been keeping headache diaries; (5) patients who had first agreed to have three-month CGRP-mABs, but had only one-time CGRP-mABs. We did not intend to perform one-time administration of CGRP-mABs. We first explained that these CGRP-mABs are used for at least three months continuously. However, patients could not visit us for the second CGRP-mABs administration due to the coronavirus disease 2019 pandemic and refraining from infection [9], holidays, business, bad weather conditions causing traffic stops, and unavailability to online telemedicine [10]. We investigated patients who received only the first-time CGRP-mABs but not second and subsequent administration. We excluded patients whom we could not ask about the efficacy of CGRP-mABs at one and three months.

From the 85 CGRP-mABs-treated patients’ medical records between April 2021 and December 2022, we retrospectively investigated eight migraine patients aged from 19 to 63 years who were treated with one-time administration of CGRP-mABs at our headache-specialized outpatient department. All eight patients met the inclusion criteria. The oral prophylactic medication, which had been taken before CGRP-mABs administration, continued for three months or tapered off after the one-time administration of CGRP-mABs unless it had adverse effects. We tapered off the prophylactic medications if the patients had no headaches in the one evaluated month. The headache diagnosis was based on the International Classifications of Headache Disorder third edition [11]. Chronic migraine (CM), episodic migraine (EM), tension-type headache (TTH), and medication-overuse headache (MOH) were diagnosed. We obtained written informed consent for this study from all patients or their families. This retrospective study was performed following the Declaration of Helsinki. The study approval was obtained from the Itoigawa General Hospital Ethics Committee (approval number: 2021-4).

Clinical variables and outcomes

We collected patients’ characteristics, such as age, sex, and previous oral prophylactic medications. Clinical data reported by paper-based or electronic headache diaries were used. Monthly headache days (MHD) and monthly acute medication intake days (AMD) were defined as the monthly values over the respective observation period of 30 days. Acute medications were non-steroidal anti-inflammatory drugs, triptans, or lasmiditan. The Head Impact Test-6 (HIT-6) [12] score was also investigated over the observation period. The outcomes were defined as the changes in MHD, AMD, and HIT-6 scores before treatment and after one and three months. If the patient did not come to the hospital, we followed up by telephone or online telemedicine [10]. We defined the HIT-6 score under 50 as therapeutically effective.

Statistical analysis

Results were presented as the median (range). A Friedman’s test and a subsequent Wilcoxon’s test were performed to compare MHD, AMD, and HIT-6 scores before treatment and after one or three months. We conducted these analyses using SPSS software version 28.0.0 (IBM Corp., Armonk, NY, USA). A two-tailed p-value <0.05 was considered statistically significant. Correction for multiple comparisons in each test was applied, but we did not apply it throughout the study [13].

## Results

Table 1 shows the characteristics of the eight migraine patients treated with one-time administration of CGRP-mABs.

**Table 1:** Patient characteristics AMD: monthly acute medication intake days; CGRP-mABs: calcitonin gene-related peptide-related monoclonal antibodies; CM: chronic migraine; EM: episodic migraine; HIT-6: Headache Impact Test-6; MHD: monthly headache days; MOH: medication-overuse headache; TTH: tension-type headache

Number	CGRP-mABs	Age	Sex	Diagnosis	Medication	Zero-month MHD	Zero-month AMD	Zero-month HIT-6	One-month MHD	One-month AMD	One-month HIT-6	Three-month MHD	Three-month AMD	Three-month HIT-6	Follow-up
1	Galcanezumab	19	M	EM+TTH	Valproic acid	30	4	65	3	2	49	0	0	36	Three months. Taperd off valproic acid
2	Fremanezumab	33	W	EM+TTH+MOH	Goreisan, goshuyuto. Amitriptyline caused nausea	30	30	60	5	5	42	2	2	40	Five months. Continued goreisan and goshuyuto
3	Fremanezumab	38	W	CM+MOH	Valproic acid	30	30	78	1	1	44	2	0	48	Aggravation at eight months and developed MOH (MHD 30 days, AMD 30 days, HIT-6 score; 72). Restarted fremanezumab
4	Galcanezumab	46	M	EM+TTH+MOH	Goreisan, amitriptyline	30	30	64	10	3	44	20	20	58	One month. Did not want to restart galcanezumab but continued amitriptyline. Referred to psychiatry as somatic symptom disorder
5	Galcanezumab	47	M	CM+MOH	Goreisan. Amitriptyline caused nausea	30	30	60	30	10	57	30	11	56	One month. Did not want to restart galcanezumab or any other prophylaxis due to business
6	Fremanezumab	55	W	EM	Goreisan. Propranolol caused bronchial asthma	4	4	54	1	0	46	0	0	36	Eight months. Tapered off goreisan.
7	Fremanezumab	61	W	EM+TTH	Valproic acid, goreisan	10	1	50	4	1	45	0	0	38	Six months. Continued goreisan, valproic acid
8	Fremanezumab	63	W	EM+TTH	Propranolol and amitriptyline	30	5	61	4	0	52	0	0	48	Eight months. Continued propranolol and amitryptiline

A total of five women and three men were included. The median age was 46.5 (19-63) years. Of the eight patients, one had EM, three had EM+TTH, two had CM+MOH, and two had EM+TTH+MOH. Five patients received one-time administration of 225 mg fremanezumab, and three received 240 mg galcanezumab. Other details are described in Table 1. Five of the eight patients took Japanese herbal* Kampo *medicine [14,15] as prophylaxis before CGRP-mABs treatment.

Of the eight patients, six (75.0%) experienced therapeutic effectiveness one month after the one-time administration of CGRP-mABs (Patients 1, 2, 3, 4, 6, 7). Five of the six maintained the therapeutic effect until three months (Patients 1, 2, 3, 6, 7), but one had aggravation (Patient 4). Patient 3 developed MOH at eight months and restarted monthly 225 mg fremanezumab. As a result, six (75.0%) patients reached or remained in good condition three months after the one-time administration of CGRP-mABs (Patients 1, 2, 3, 6, 7, 8). On the other hand, two of the eight (25%) did not feel therapeutic effects (Patients 4, 5). Patient 4 was finally referred to psychiatry for having somatic symptom disorder. Patient 5 used galcanezumab and oral prophylaxis only for one month. He could not keep a headache diary with poor adherence. There were no side effects of CGRP-mABs. All patients continued previously used oral prophylaxis during the observational period.

**Figure 1:**
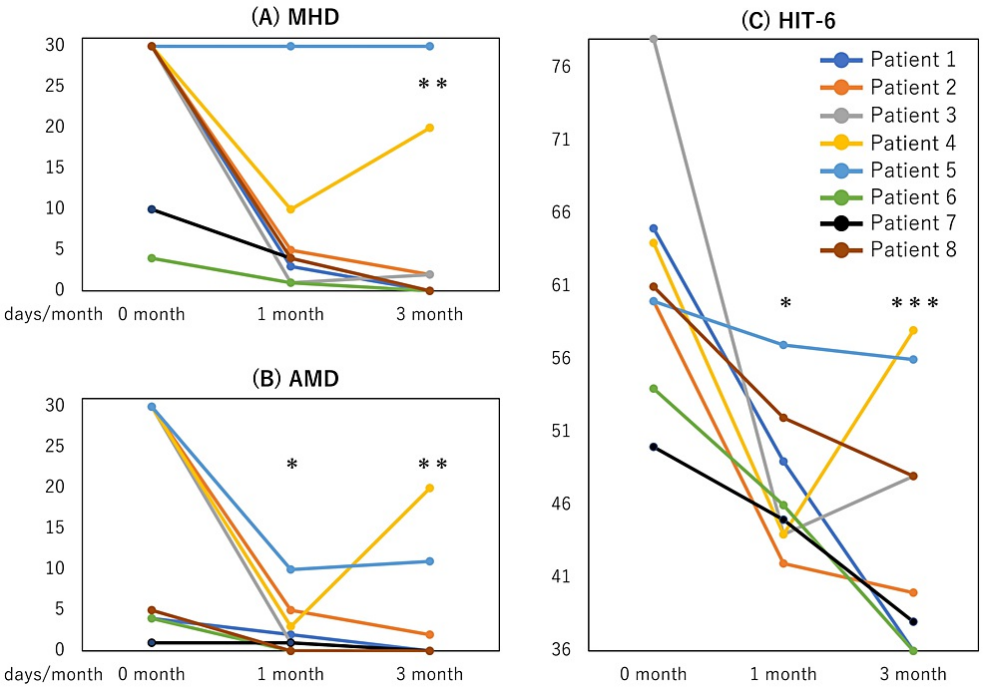
Therapeutic effects. Chronological changes of monthly headache days (MHD) (A), monthly acute medication intake days (AMD) (B), and Headache Impact Test-6 (HIT-6) scores (C) after one-time administration of galcanezumab 240 mg or fremanezumab 225 mg (zero months). *: p < 0.05; significantly decreased compared to zero months adjusted by Bonferroni’s correction. **: p < 0.01; ***: p < 0.001.

Figure 1 shows the therapeutic effects. The median MHD before, one, and three months after treatment were 30 (4-30), 4 (1-30), and 1 (0-30), respectively (Figure 1A). Those for AMD were 17.5 (1-30), 1.5 (0-10), and 0 (0-20), respectively (Figure 1B). Those for HIT-6 scores were 60.5 (50-78), 45.5 (42-57), and 44 (36-58) (Figure 1C), respectively. Not MHD but AMD and HIT-6 scores significantly improved at one month (p = 0.073, p = 0.037, and p = 0.037, respectively). Significant reductions in MHD, AMD, and HIT-6 scores were observed three months after the initial administration of CGRP-mABs (p = 0.008, p = 0.005, and p < 0.001, respectively).

## Discussion

Here, we described eight migraine patients who were treated with one-time administration of galcanezumab 240 mg or fremanezumab 225 mg in combination with oral prophylaxis. Of the eight patients, six (75.0%) experienced or maintained therapeutic effectiveness at three months despite the one-time administration of CGRP-mABs. There were no side effects of CGRP-mABs. This is the first case series of one-time administration of CGRP-mABs, and our results suggested that one-time use of CGRP-mABs may be a new treatment option combined with oral prophylaxis.

The randomized placebo-controlled studies [7,8] showed the immediate effects of CGRP-mABs in reducing the monthly migraine days in the first month after administration. This rapid effect is a strength of CGRP-mABs [16], considering that oral prophylaxis needs at least two to three months to establish its effects. Despite its rapidity and effectiveness, the main reason many patients are hesitant to receive CGRP-mABs is the drug price. Some studies have shown that long-term CGRP-mABs administration gradually increases efficacy [17,18]. However, considering the ineffectiveness possibility and the cost, it is understandable that some patients are hesitant about administering CGRP-mABs for three months in a row.

The time to reach peak blood concentration and biological half-life of 240 mg galcanezumab are 124 hours and 26 days, respectively. Those of 225 mg fremanezumab are seven days and 33.6 days, respectively. Theoretically, the efficacy of one-time administration becomes weaker after one month. However, six of our eight patients experienced or maintained therapeutic effects until three months. We proposed a hypothesis as a reason for why one-time use of CGRP-mABs had a three-month-lasting therapeutic effect.

CGRP-mABs exert their effects primarily in and around the trigeminal nerve, and most of them cannot pass the blood-brain barrier. In other words, CGRP-mABs act mainly on peripheral nerves. On the other hand, valproic acid, amitriptyline, and propranolol can partly pass the blood-brain barrier. Japanese herbal *Kampo* medicines are also thought to act partly in the central nervous system [19]. These oral prophylactic medications act mainly in the central nervous system. Possibly, the effects on each of the central and peripheral nerves were synergetically exerted by CGRP-mABs and oral prophylaxis.

The most frequently reported trigger for migraine is stress [20]. On the other hand, migraine itself brings stress [1]. The patients’ experience of the rapid and robust prophylactic action of CGRP-mABs may have transformed their lives and freed them from stress. This may have allowed them to break free from the negative spiral of headache-causing stress, which is caused by another headache. Suppose the efficacy of a single dose is established, in that case, CGRP-mABs could be used only once when the patients have an appointment that they do not want to have migraine attacks, such as a trip, a wedding party, an academic examination, or an important job. It could also be used temporarily only during migraine-attack-frequent seasons, such as rainy or busy seasons. Further real-world data on the one-time use of CGRP-mABs are needed.

This study had some limitations. The present results were based on a small number of cases at a single institution in Japan. Therefore, it remains unknown whether the results will be similar for migraine patients globally. Our results were preliminary, and this study needs a larger sample size to reach a more definite conclusion. CGRP-mAB injections may have triggered the patients to keep a headache diary and think about their health more carefully. It is also possible that other prophylactic drugs may have had a delayed effect. Further investigation with a large population is needed.

## Conclusions

Galcanezumab and fremanezumab were evaluated at 12 weeks in randomized control trials. However, there are no reports on the effectiveness of one-time use evaluated at one and three months for migraine prevention. This is the first case series of one-time administration of CGRP-mABs. The eight migraine patients were treated with one-time administration of galcanezumab 240 mg or fremanezumab 225 mg in combination with oral prophylaxis. Six (75.0%) of the eight patients experienced or maintained therapeutic effectiveness at three months despite the one-time administration of CGRP-mABs without any side effects. Our results suggested that one-time use of CGRP-mABs may be a new treatment option combined with oral prophylaxis. Further investigation of the one-time use of CGRP-mABs is required.

## References

[1] Matsumori Y, Ueda K, Komori M (2022). Burden of migraine in Japan: results of the ObserVational Survey of the Epidemiology, tReatment, and Care Of MigrainE (OVERCOME [Japan]) study. Neurol Ther.

[2] Sakai F, Igarashi H (1997). Prevalence of migraine in Japan: a nationwide survey. Cephalalgia.

[3] Katsuki M, Yamagishi C, Matsumori Y (2022). Questionnaire-based survey on the prevalence of medication-overuse headache in Japanese one city-Itoigawa study. Neurol Sci.

[4] Takeshima T, Ishizaki K, Fukuhara Y (2004). Population-based door-to-door survey of migraine in Japan: the Daisen study. Headache.

[5] Katsuki M, Matsumori Y, Kawahara J (2022). School-based online survey on chronic headache, migraine, and medication-overuse headache prevalence among children and adolescents in Japanese one city -Itoigawa Benizuwaigani Study. ResearchGate Preprint.

[6] Katsuki M, Kawahara J, Matsumori Y (2022). Questionnaire-based survey during COVID-19 vaccination on the prevalence of elderly's migraine, chronic daily headache, and medication-overuse headache in one Japanese City-Itoigawa Hisui study. J Clin Med.

[7] Förderreuther S, Zhang Q, Stauffer VL, Aurora SK, Láinez MJ (2018). Preventive effects of galcanezumab in adult patients with episodic or chronic migraine are persistent: data from the phase 3, randomized, double-blind, placebo-controlled EVOLVE-1, EVOLVE-2, and REGAIN studies. J Headache Pain.

[8] McAllister P, Cohen JM, Campos VR, Ning X, Janka L, Barash S (2022). Impact of fremanezumab on disability outcomes in patients with episodic and chronic migraine: a pooled analysis of phase 3 studies. J Headache Pain.

[9] Katsuki M, Matsumori Y, Kawahara J (2023). Headache education by leaflets distribution during COVID-19 vaccination and school-based on-demand E-learning -Itoigawa Geopark Headache Awareness Campaign. Research Square.

[10] Katsuki M (2022). The first case series from Japan of primary headache patients treated by completely online telemedicine. Cureus.

[11] (2018). Headache Classification Committee of the International Headache Society (IHS). The International Classification of Headache Disorders, 3rd edition. Cephalalgia.

[12] Kosinski M, Bayliss MS, Bjorner JB (2003). A six-item short-form survey for measuring headache impact: the HIT-6. Qual Life Res.

[13] Rothman KJ (1990). No adjustments are needed for multiple comparisons. Epidemiology.

[14] Katsuki M, Kawamura S, Kashiwagi K, Koh A (2021). Medication overuse headache successfully treated by Japanese herbal Kampo medicine, Yokukansan. Cureus.

[15] Katsuki M, Kashiwagi K, Kawamura S, Koh A (2022). The efficacy of Japanese herbal Kampo medicine as an acute and prophylactic medication to treat chronic daily headache and medication overuse headache:-single arm retrospective study. Cureus.

[16] Igarashi H, Shibata M, Ozeki A, Day KA, Matsumura T (2021). Early onset and maintenance effect of galcanezumab in Japanese patients with episodic migraine. J Pain Res.

[17] Stauffer VL, Dodick DW, Zhang Q, Carter JN, Ailani J, Conley RR (2018). Evaluation of galcanezumab for the prevention of episodic migraine: the EVOLVE-1 randomized clinical trial. JAMA Neurol.

[18] Blumenfeld AM, Stevanovic DM, Ortega M (2020). No "wearing-off effect" seen in quarterly or monthly dosing of fremanezumab: subanalysis of a randomized long-term study. Headache.

[19] Katsuki M, Kawamura S, Kashiwagi K, Koh A (2021). Medication overuse headache successfully treated by three types of Japanese herbal Kampo medicine. Cureus.

[20] Kelman L (2007). The triggers or precipitants of the acute migraine attack. Cephalalgia.

